# Mechanism and Catalytic Site Atlas (M-CSA): a database of enzyme reaction mechanisms and active sites

**DOI:** 10.1093/nar/gkx1012

**Published:** 2017-11-02

**Authors:** António J M Ribeiro, Gemma L Holliday, Nicholas Furnham, Jonathan D Tyzack, Katherine Ferris, Janet M Thornton

**Affiliations:** European Molecular Biology Laboratory, European Bioinformatics Institute, Wellcome Trust Genome Campus, Hinxton, Cambridge CB10 1SD, UK; Department of Pathogen Molecular Biology, London School of Hygiene and Tropical Medicine, Keppel Street, London WC1E 1HT, UK

## Abstract

M-CSA (**M**echanism and **C**atalytic **S**ite **A**tlas) is a database of enzyme active sites and reaction mechanisms that can be accessed at www.ebi.ac.uk/thornton-srv/m-csa. Our objectives with M-CSA are to provide an open data resource for the community to browse known enzyme reaction mechanisms and catalytic sites, and to use the dataset to understand enzyme function and evolution. M-CSA results from the merging of two existing databases, MACiE (**M**echanism, **A**nnotation and **C**lassification **i**n **E**nzymes), a database of enzyme mechanisms, and CSA (**C**atalytic **S**ite **A**tlas), a database of catalytic sites of enzymes. We are releasing M-CSA as a new website and underlying database architecture. At the moment, M-CSA contains 961 entries, 423 of these with detailed mechanism information, and 538 with information on the catalytic site residues only. In total, these cover 81% (195/241) of third level EC numbers with a PDB structure, and 30% (840/2793) of fourth level EC numbers with a PDB structure, out of 6028 in total. By searching for close homologues, we are able to extend M-CSA coverage of PDB and UniProtKB to 51 993 structures and to over five million sequences, respectively, of which about 40% and 30% have a conserved active site.

## INTRODUCTION

Enzymes are the macromolecules that catalyze the chemical reactions of life. The study of enzymes draws from the fields of biochemistry, genomics, protein structure, organic chemistry, computational chemistry, thermodynamics, and metabolomics, amongst others. As discussed below, current literature and biological databases with enzyme information mirror this diversity.

Enzymes are one of the most common products of the translation of genetic information. Protein sequence databases, most notably UniProtKB and its manually curated subset, Swiss-Prot, capture protein sequence data, including that for enzymes (1). Sequence is but a small part of understanding how enzymes work, but due to the explosion of sequencing data (there are, at the moment, more than 89 million sequences in UniProtKB across 141 511 proteomes), it is an essential tool if one wants to extend current knowledge throughout the tree of life. Currently, 47.2% (262 310) of the 555 426 reviewed Swiss-Prot entries and 13.5% (12 050 143) of the complete UniProtKB dataset are annotated as enzymes, the latter figure primarily reflecting incomplete knowledge and annotation.

The three-dimensional structures of enzymes, as deposited in the wwPDB (2), are a much richer source of information for understanding function than sequence alone. At the moment, the wwPDB contains 133 397 structures, 45.9% (61 168) of which annotated as enzymes (3). The number of available enzyme structures is not as vast as the number of sequences (and many sequences have more than one crystal structure), which is an important limitation. Without structure, elucidation of a specific mechanism is much more difficult, because the identification of catalytic and binding residues can only proceed through indirect means. Structure can also help elucidate mechanisms that have been debated for many years (e.g. lysozyme) (4). Besides revealing functional mechanisms, structure also allows the identification of distant evolutionary links, since structural domains, like the ones defined in CATH (5) and SCOP (6), are more conserved than sequence (7,8). The version of CATH (v4.1) used here contains 2737 homologous superfamilies, 46% (1268) of these are annotated with at least one EC number and 845 are found in M-CSA.

The chemical reaction is the complete chemical transformation that the enzyme performs, and is often described with an Enzyme classification (EC) number (9). Currently, there are 6,028 reactions in the EC classification (10). In a strict sense, the EC classifies enzyme reactions, not enzymes, but each EC number is often linked to all the enzyme sequences that catalyse that reaction, even if the enzyme mechanisms differ (e.g. there are three cases of evolution for the chloroperoxidase reaction: EC 1.11.1.10). The primary EC database is hosted in ExplorEnz (11) and replicated by IntEnz (12) and ExPASy ENZYME (10). Other databases of enzyme reactions include more annotation than just the EC data. RHEA (13) describes reactions using ChEBI (14) identifiers to define reactants and products, and provides an RXN file for each reaction. The Enzyme and Reaction subsets of the KEGG database (15) define reactions using molecules from KEGG Compound, and these are also linked to their metabolic database, KEGG Pathway. Databases with functional information, such as BRENDA (16) and Sabio-RK (17), include kinetic data across enzymes of different organisms, mutants, and in different chemical conditions. Enzymes are also crucial components of metabolism databases. Examples of these include MetaCyc (18), which contains pathways for several organisms, ReconMap (19), which focus on human metabolism, and the already mentioned KEGG Pathway. In M-CSA, the overall reaction is primarily described as a list of reactants and products and is also assigned an EC number (to the most detailed level possible).

These databases tell us about enzyme function, and where this function fits in the overall scheme of cells, but they do not tell us how enzymes work. This is the unique contribution of the M-CSA (which stands for **M**echanism and **C**atalytic **S**ite **A**tlas) and its parent databases, MACiE (**M**echanism, **A**nnotation and **C**lassification **i**n **E**nzymes), and CSA (**C**atalytic **S**ite **A**tlas). Data about the reaction mechanisms of enzymes are found throughout the literature in several forms. Catalytic residues are usually identified by noting their conservation in related homologues, and by testing the lack of enzyme activity when these residues are mutated. Measuring enzyme activity under several biochemical conditions can also be insightful. For example, simply changing the pH may reveal the protonation states most conducive to catalysis. Protein structures, which can be found in the wwPDB, reveal the position of the active centre and the 3D disposition of catalytic residues. Structures that contain the substrate, the products or a similar molecule are yet more informative. Computational chemistry studies of enzyme mechanisms have been gaining traction over the last decade, propelled by new algorithms and increasing computational power. QM/MM models, in particular, which combine a QM description of the active center—usually using DFT methods—with a Molecular Mechanics description of the rest of the enzyme have the potential to provide a complete prediction of the mechanistic process in atomic detail (20,21).

M-CSA, as well as MACiE (22–24) and CSA (25–27), were created to capture and organize these and other kinds of mechanistic data available in the literature, and to make them available in a standardised and computer readable format for the community. Additionally, these datasets have been helpful to explore overall themes related to enzyme mechanisms such as the evolution of new chemical function and the roles of specific catalytic residues, cofactors, and metal ions in the chemistry of life (28–30). M-CSA includes annotation for the complete catalytic reaction and also for each step of that reaction, which includes the curly arrow description of the stepwise chemical reaction mechanism, the role of each catalytic residue and any cofactors, as well as the primary literature that supports such data. We also provide annotation for the associated protein sequences and structures, and when appropriate, we link to the databases mentioned above. M-CSA is complementary to other mechanism databases like the Structure-Function Linkage Database (SFLD) (31), which annotates diverse enzyme superfamilies (groups of enzymes that are evolutionarily related and perform a disparate set of overall chemical transformations utilising conserved chemical components), and EzCatDB (32), which uses a hierarchic classification of catalytic mechanisms (RLCP) to classify reaction mechanisms.

M-CSA represents a complete overhaul of the MACiE and CSA databases and websites, as well as an update of the content. We merged the two databases together due to their similarities and to avoid duplication of effort. As of September 2017, the updated database contains 423 manually curated entries with detailed reaction mechanisms and 538 manually curated entries where likely catalytic residues have been identified, but the complete mechanism is not known. We extend this annotation to over five million homologues sequences using the UniProtKB reference dataset, and to 51,993 homologous PDB structures. Below, we describe in more detail the features of the M-CSA website and the main differences with respect to the parent databases.

## CONTENT UPDATE

### Merging CSA and MACiE

MACiE (22–24) was a database of enzyme mechanisms that primarily annotated individual mechanism reaction steps with 2D curly arrow schemes, the roles of catalytic residues and cofactors, and a text description. Other annotation in MACiE included the overall reaction and links to PDB, CATH, UniProtKB and other databases. The CSA (Catalytic Site Atlas) (26,27), on the other hand, was a manually curated database of enzyme catalytic residues. Each entry in CSA contained a list of catalytic residues with their chemical functions annotated, together with the literature evidence, the overall reaction, and a reference PDB entry. Additionally, each entry included a list of homologous PDB structures which greatly increased the coverage of the dataset. The annotation of CSA overlapped with the annotation of MACiE, and there had been some efforts in the past to standardize the two databases using the EMO ontology and controlled vocabulary (27). The only annotation that MACiE lacked, in comparison with CSA, was the function of each catalytic residue at the overall reaction level and the PDB homologues associated with each entry. Apart from that, the CSA annotation was essentially a subset of the MACiE annotation.

The integration of the two databases accomplished here avoids the future duplication of effort and resources which, among other advantages, will make future updates easier. In M-CSA we have two kinds of entries, ‘catalytic site’ entries that represent the level of annotation associated with CSA entries, and ‘detailed mechanism’ entries that represent the level of annotation associated with MACiE entries. Both types of entries are now created and updated using the same web interface, and it is easy to promote ‘catalytic site’ entries to ‘detailed mechanism’ entries by just adding the missing mechanistic annotation.

While most of the data migration to the new database was automated, part of the merging process was performed manually. Besides the addition of the overall function field for each residue in MACiE, there was some duplication between CSA and MACiE that had to be removed. Duplication was identified initially at the EC, structure and sequence levels, although not all entries that share one of these are duplicates, because the same EC reaction can be catalysed by different catalytic sites/mechanism, and the same structure or sequence can catalyse more than one reaction. Manual curation was necessary to identify true duplicates, and to decide which fields to keep from each entry, if these were inconsistent. A list of current entries with the same EC or UniProtKB ID is given in the SI. During the data integration process, we found 303 CSA entries that were duplicates of existing MACiE entries and 127 CSA entries that were determined to be duplicates of other CSA entries.

### Data migration and literature review

Over the years, new studies are published that invalidate, or complement, previous mechanism proposals. In the previous versions of MACiE we could only save a single mechanism proposal for each entry. We now store all the mechanism proposals we can find for each enzyme. We rate these alternatives from one to three stars where: one star denotes a proposed mechanism which has been disproved by more recent data; two stars denote a proposed mechanism that does not explain all the evidence and three stars denotes a proposed mechanism that is consistent with all existing evidence. Of the 423 detailed mechanism entries in the database, 50 entries have more than one mechanism proposal, and 10 have more than two.

Besides adding new mechanism proposals, and new literature references that support new and existing mechanisms, other changes to the curated data included: the update of text fields where necessary; the selection of more relevant PDB representatives or UniProtKB IDs as reference proteins; the addition of new reactions to the same entry (where the same mechanism at the same reaction centre works on different reactants); linking M-CSA reactions to KEGG and RHEA reactions, and lastly, selecting a primary literature reference for each mechanism proposal. In the old database, schemes of reaction were only available as RXN files, which did not include the electron arrows. Arrows were added on top of a static .gif image. In the new version of the website we use MarvinJS to draw the reaction schemes, which allow us to embed the curly arrows in the same file.

At the time of the release all entries coming from both the CSA and MACiE have been brought up to the same standard, where the annotations for the two types of entries are equivalent, except for the lack of a mechanism in catalytic site entries. Additionally, a complete literature review of all the entries in M-CSA is in progress.

### New statistics

The statistics page in the website provide real time analysis of the database. These plots are interactive and some of them link to the appropriate enzyme entries. One example is shown below (two more examples are given in the SI), but we encourage the reader to try others online. We plan to expand these in the future as we further study the M-CSA dataset.

**Figure 1.**
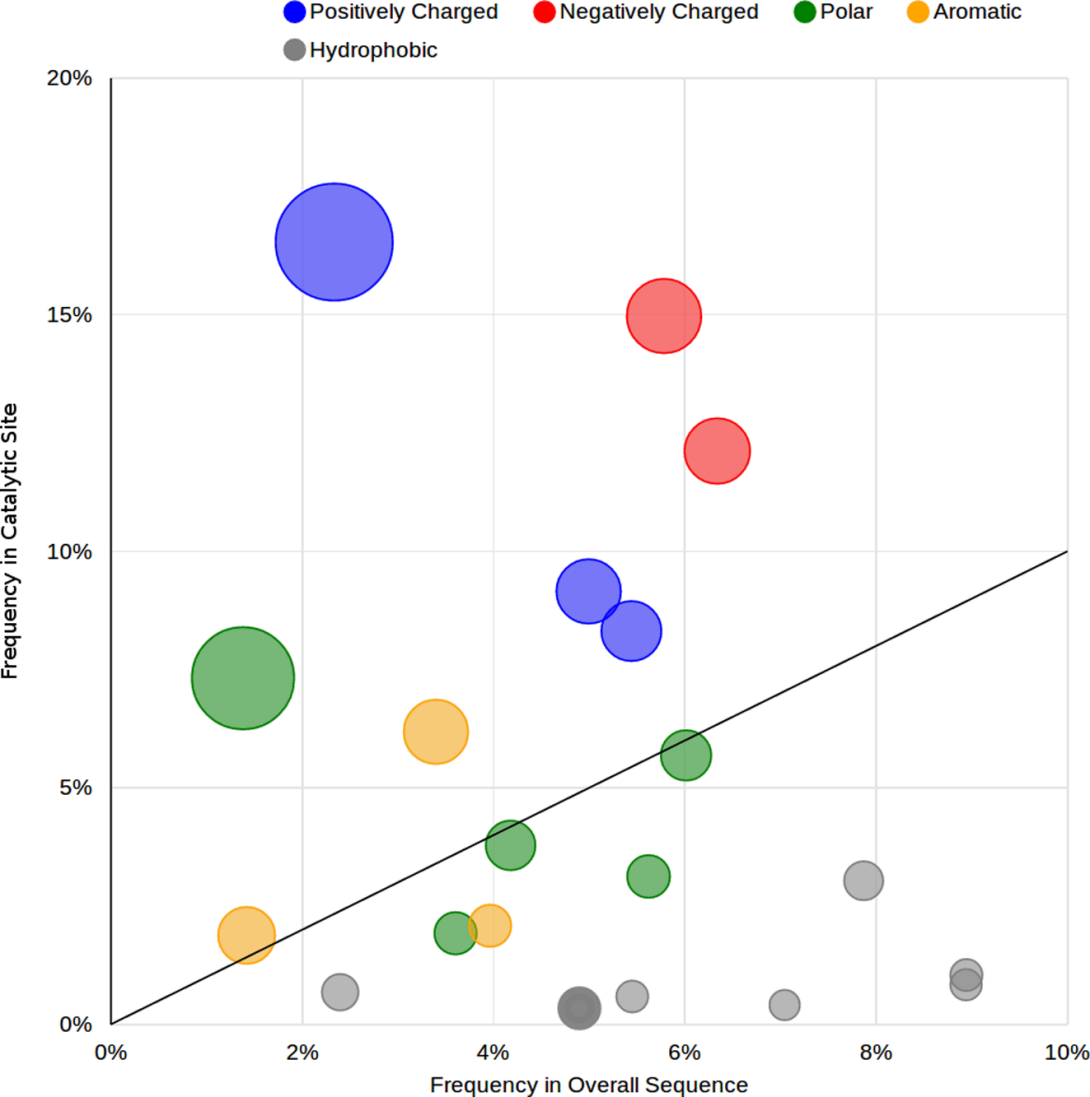
Frequencies and catalytic propensity of the 20 amino acids as they appear in current the M-CSA dataset.

The catalytic propensity of an amino acid captures how frequently an amino acid is involved in catalysis compared with random chance, i.e. the ratio between the percentage of that residue that is catalytic (in the whole M-CSA dataset) and the percentage of that residue in the protein sequences. Figure 1 plots the frequency of the 20 amino acids in the active site of all entries in MACiE against their frequency in the complete protein sequence. The catalytic propensity is indicated by the size of the circle (bigger means higher propensity) and the position in the plot (near the upper left corner means higher propensity). If the propensity is <1, then the propensity for that residue to be catalytic is less than expected by chance, and if it is >1, then the residue is more catalytic than might be expected. Colours indicate the type of amino acid. The distribution is clear: hydrophobic amino acids very rarely have a catalytic role, even though the four most frequent amino acids in these proteins are hydrophobic (alanine, leucine, glycine and valine). Charged amino acids, together with Cysteine, have the highest propensity. Histidine is seven times more likely to appear as a catalytic amino acid than expected from a random distribution.

## FEATURES OF THE NEW WEBSITE AND DATABASE

### Home and entry pages

All the pages in the new website were designed from scratch. We try to present all the information captured in the database in a clear and intuitive manner. The user can navigate the website using a navigation bar that is always visible. Searching is also available from the top header in all pages.

The home page contains a brief description of M-CSA, additional navigation links, citation information, and a brief text description of the main statistics. The most prominent part of the homepage is a frame that summarizes the data of an example entry, to give new users an immediate feel for the information contained in M-CSA. This frame contains the identification of the example enzyme, some links to other databases, the description of the enzyme, and an image of one step of the mechanism. The user can click on the image to be taken to the respective entry page.

The entry page contains all the data associated with each enzyme mechanism in the database. A 3D Litemol window shows the reference PDB structure for this enzyme, where the ‘show active centre’ button can be used to zoom into the catalytic residues which are automatically highlighted, as well as any reactants and/or cofactors that happen to be in the structure. In the bottom part of the entry page, in the mechanism box, there is now a new table, similar to one available for the CSA, that shows the overall role of each catalytic residue in the reaction. Reaction schemes are shown on separate tabs, together with the role of the residues in each step. This page also links to the PDB and UniProt sequence homologues pages, where the user can browse the homologues and check the conservation of the catalytic residues in these sequences. Homologous sequences were found by running every M-CSA reference sequence and PDB sequence against the UniProfRef and PDB databases, respectively, using HMMER (33).

### Browse, search and download pages

**Figure 2.**
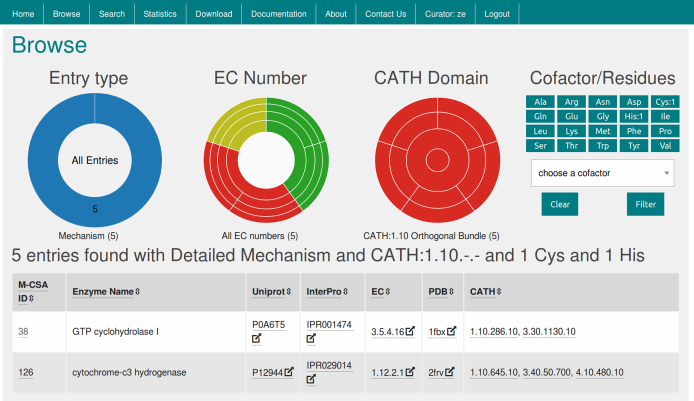
The browse page. Example of how to find all M-CSA entries with detailed mechanism, CATH:1.10.-.- and at least one His and one Cys in the catalytic site. The bottom three rows of the results table are omitted in the screenshot.

The browse page gives the user an overall view of the database. The default view shows a table with all the entries. This table can be sorted by any of UniProtKB, PDB, EC and CATH identifiers. The doughnut and sunburst charts, together with the residues and cofactors selectors, allow the user to query the database in graphical way. For example, Figure 2 shows how the user can query for all the entries with detailed mechanism, CATH 1.10.-.- (Orthogonal Bundle) and with at least a cysteine and a histidine as catalytic residues.

The statistics pages can also be used for browsing. Most of the plots have interactive elements that the user can click to retrieve relevant M-CSA entries or affect what is being shown on other plots. For example, in the Residues page, the user can click on a residue and then on a role, to be shown a table with all the entries where that residue has the selected role.

The search page contains a single search box and several buttons that can be toggled to select which tables to search in the database. Most fields and tables in the database can be searched in this manner. The search functionality can also be accessed by the search box situated in the main header, which is visible across the website. In that case, the default search fields will be used (enzyme name, EC, PDB, and UniProtKB identifiers). The results of the search are presented in a table under the search box.

We provide flat files with the data in M-CSA in the downloads page. Currently, the more complete file provides the reference PDB and UniProtKB sequences for each entry, as well as the EC number for the reference reaction and a list of reactants and products. It also contains a list of all the catalytic residues and cofactors together with their annotated roles for each entry. We also provide flat files in the same format as the old CSA website, for users that have incorporated that in their workflow.

### Curator pages

The curator pages can be used to edit the database, and make the changes immediately visible across the website. Remote submission for new entries is now possible, through these pages. Additional automation and data checks (examples in SI), in relation to previous databases facilitate curation and minimize the introduction of errors. The documentation pages describe how to add new entries to the database using the curator tools.

We use the ChemAxon MarvinJS plugin (www.chemaxon.com) to draw the chemical mechanistic steps and save these in the ChemAxon Marvin Document (MRV) file type. Besides the molecules’ 2D coordinates, the MRV file type also saves the position of electron flow arrows, which is essential for our purposes.

The process of entering a new entry into the database is as follows. Curators are assigned a password protected account which allows them to create and edit their own entries. After the edition of a new entry is finished, the curator flags that entry as complete. A member of staff, which is a curator with more privileges, will then review that entry and flag it so it can be shown on the public part of website. At this point, the ownership of the entry is changed and the original curator cannot edit this entry anymore. Only staff curators can edit or delete public M-CSA entries.

### Technology

The new website has been implemented using the Django Web Framework v1.10 (djangoproject.com) together with a PostgreSQL database (www.postgresql.org). The database schema is shown in the SI. Python is the only language used on the server side. Custom JavaScript and CSS, apart from the JS plugins and the EBI template, are used sparingly on the client side. We use LiteMol (34), a JavaScript plugin initially developed for PDBe (35), to show PDB structures and highlight the catalytic residues and substrate. In the curator pages, we use the ChemAxon MarvinJS plugin v17.15.0 (www.chemaxon.com) to draw chemical schemes of reaction and chemical compounds that are not available in ChEBI, although curators are encouraged to add compounds directly to ChEBI, instead, if they have access. Reaction Decoder (36), the atom mapping tool that is part of EC-Blast (37), is used to map RXN files, in order to calculate bond changes at the overall reaction level. All plots on the website, including the sunbursts plots in the browse page and all the plots in the statistics pages are rendered by NVD3 (nvd3.org), a D3.js (d3js.org) extension.

## FUTURE DEVELOPMENTS

We hope to continue updating M-CSA in the future. In 2011, the last time these databases were updated, MACiE and the CSA covered over 70% of the available EC numbers with 3D-structures (a proxy for the number of cases with an available mechanism). Since 2011, the number of EC numbers with sequences and 3D structures has more than doubled (1196–2793). However, it cannot be assumed that because there is a structure, the mechanism is known. We do not currently have a good estimate of how many more mechanisms are available since 2011, although a conservative estimate would be double, in line with the increase of EC numbers with a 3D structure.

We are currently working on a 3D representation of the mechanism, for which we are building 3D models guided by the 2D schemes and some simple structural constraints. Ideally, we would like to use structures coming from computational chemistry studies, but these are not generally available. We encourage computational chemists to send us their models of enzyme mechanisms for inclusion in the database.

Since curation is now simpler and can be done through the website, we encourage enzymologists to submit new reaction mechanisms, especially for reactions not currently captured in M-CSA. In turn, and coupled with the rest of the analysis we want to perform, these users would be able to compare their proposed mechanism with others and see how it fits into the overall landscape of catalysis. For example, we could detect that a catalytic residue is doing a never-seen role, which could indicate that something is wrong with the mechanism or that the user has found some new chemistry.

We also want to use more protein structural tools to improve the database. At the moment, we are finding homologous proteins through sequence search, but structural alignments like those provided by CATH-tools (38), can uncover more distant relatives. Additionally, 3D template search using the catalytic residues (39,40) can help us find similar active sites in other structures, which may not be apparent from the one-dimensional sequence similarity.

## Supplementary Material

Supplementary Data
