# Supplementary material for: Mechanism and Catalytic Site Atlas (M-CSA): a database of enzyme reaction mechanisms and active sites
Source: Nucleic Acids Res. 2017 Nov 2;46(Database issue):D618–23. doi: 10.1093/nar/gkx1012 (PMC5753290; doi:10.1093/nar/gkx1012)
Supplement: Supplementary Data [file gkx1012_supp.pdf]

Supporting Information

Mechanism and Catalytic Site Atlas (M-CSA): A database of enzyme reaction mechanisms and active sites.

Antonio J. M. Ribeiro,[1,†,*] Gemma L. Holliday,[1,†] Nicholas Furnham,[2] Jonathan D. Tyzack,[1] Katherine Ferris,[1] Janet M. Thornton[1]

[†]These authors contributed equally to the paper as first authors.

[1]European Molecular Biology Laboratory, European Bioinformatics Institute, Wellcome Trust Genome Campus, Hinxton, Cambridge CB10 1SD, UK.

[2]Department of Pathogen Molecular Biology, London School of Hygiene and Tropical Medicine, Keppel Street, London, WC1E 1HT, UK.

*To whom correspondence should be addressed. Email: ribeiro@ebi.ac.uk

Contents

1. List of M-CSA Entries with the same EC numbers 1
2. List of M-CSA Entries with the same reference UniProt ID 15
3. Data checks and automation in curator pages 16
4. Other examples from the statistics pages 16

1. List of M-CSA Entries with the same EC numbers

1.1.1.1

m-csa:255 - alcohol dehydrogenase (SDR type)

m-csa:256 - alcohol dehydrogenase (class I)

m-csa:464 - alcohol dehydrogenase (class III)

m-csa:500 - alcohol dehydrogenase (class II)

m-csa:501 - alcohol dehydrogenase (class V)

1.1.1.37

m-csa:527 - malate dehydrogenase (type 1)

m-csa:526 - malate dehydrogenase (type 2)

1.2.1.2

m-csa:562 - formate dehydrogenase

m-csa:563 - formate dehydrogenase (nitrate-inducible)

m-csa:902 - NAD-dependent formate dehydrogenase

1.5.1.3

m-csa:112 - dihydrofolate reductase (bacterial)

m-csa:490 - dihydrofolate reductase (mammalian)

m-csa:752 - dihydrofolate reductase (type II)

1.6.1.2

m-csa:116 - NAD(P)+ transhydrogenase (AB-specific)

m-csa:496 - NAD(P)+ transhydrogenase (Re/Si-specific)

1.7.2.1

m-csa:4 - nitrite reductase (copper type)

m-csa:903 - nitrite reductase (cytochrome cd1 type)

1.8.4.-

m-csa:792 - protein disulfide oxidoreductase

m-csa:734 - protein disulfide oxidoreductase

1.8.4.11

m-csa:122 - protein-methionine-S-oxide reductase (MsrA)

m-csa:715 - protein-methionine-S-oxide reductase (MsrB)

1.9.3.1

m-csa:124 - cytochrome-c oxidase (AA3 type)

m-csa:735 - cytochrome-c oxidase (BA3 type)

1.11.1.5

m-csa:709 - cytochrome-c peroxidase (mono-heme type)

m-csa:749 - cytochrome-c peroxidase (di-heme type)

1.11.1.6

m-csa:573 - catalase (HPII)

m-csa:572 - catalase (manganese dependent)

1.11.1.7

m-csa:239 - peroxidase

m-csa:944 - Lactoperoxidase

1.11.1.10

m-csa:14 - chloride peroxidase (vanadate dependent)

m-csa:248 - chloride peroxidase (cofactor free)

m-csa:250 - chloride peroxidase (heme dependent)

1.11.1.18

m-csa:374 - Vanadium-dependent bromoperoxidase (organic molecule bromination)

m-csa:373 - Vanadium-dependent bromoperoxidase (organic sulfide oxidation)

m-csa:386 - Vanadium-dependent bromoperoxidase (HOBr Formation)

1.17.1.4

m-csa:139 - xanthine dehydrogenase (mammalian)

m-csa:698 - xanthine dehydrogenase (bacterial)

1.17.4.2

m-csa:140 - ribonucleoside-triphosphate reductase (class II)

m-csa:416 - ribonucleoside-triphosphate reductase (class III)

1.18.1.2

m-csa:142 - ferredoxin-NADP+ reductase (adrenodoxin-type)

m-csa:506 - ferredoxin--NADP reductase

2.1.1.43

m-csa:350 - histone-lysine N-methyltransferase (SET7/9 subfamily)

m-csa:691 - histone-lysine N-methyltransferase (Suvar3-9 subfamily)

2.2.1.6

m-csa:289 - acetolactate synthase (biosynthetic)

m-csa:722 - acetolactate synthase (catabolic)

2.3.1.48

m-csa:224 - histone acetyltransferase (HAT1 family)

m-csa:344 - histone acetyltransferase (crotonase type)

m-csa:524 - histone acetyltransferase (GCN5 family)

m-csa:525 - histone acetyltransferase (MYST family)

2.3.2.13

m-csa:149 - protein-glutamine gamma-glutamyltransferase (eukaryotic)

m-csa:761 - protein-glutamine gamma-glutamyltransferase (bacterial)

2.4.2.1

m-csa:17 - purine-nucleoside phosphorylase

m-csa:695 - purine-nucleoside phosphorylase

m-csa:375 - Purine nucleoside phosphorylase DeoD-type

2.4.2.19

m-csa:8 - nicotinate-nucleotide diphosphorylase (carboxylating)

m-csa:945 - nicotinate-nucleotide pyrophosphorylase (carboxylating) (type II)

2.4.2.36

m-csa:773 - NAD+---diphthamide ADP-ribosyltransferase

m-csa:919 - NAD+---diphthamide ADP-ribosyltransferase

m-csa:769 - NAD+---diphthamide ADP-ribosyltransferase

2.7.1.1

m-csa:696 - hexokinase (type I)

m-csa:651 - hexokinase (type II)

2.7.1.191

m-csa:514 - protein-Npi-phosphohistidine---sugar phosphotransferase (type III)

m-csa:513 - protein-Npi-phosphohistidine---sugar phosphotransferase (type II)

2.8.1.1

m-csa:153 - thiosulfate sulfurtransferase (eukaryotic)

m-csa:505 - thiosulfate sulfurtransferase (prokaryotic)

3.1.-.-

m-csa:839 - very short patch repair endonuclease

m-csa:837 - intron-encoded endonuclease I-PpoI

m-csa:530 - colicin-E3

m-csa:784 - 3'-5' exoribonuclease 1

m-csa:838 - Colicin-E7

3.1.1.-

m-csa:556 - esterase (estA)

m-csa:453 - Brefeldin A esterase

m-csa:900 - para-nitrobenzyl esterase

m-csa:733 - esterase

3.1.1.3

m-csa:218 - triacylglycerol lipase (pancreatic)

m-csa:518 - triacylglycerol lipase (EstA)

m-csa:519 - triacylglycerol lipase (Pseudomonas family)

m-csa:520 - triacylglycerol lipase (type B carboxylestrase)

3.1.1.4

m-csa:83 - phospholipase A2 (group IB)

m-csa:529 - phospholipase A2 (group group IVA)

m-csa:528 - phospholipase A2 (prokaryotic/fungal)

3.1.1.61

m-csa:337 - protein-glutamate methylesterase (CheB)

m-csa:729 - protein-glutamate methylesterase (CheD)

3.1.2.22

m-csa:522 - palmitoyl[protein] hydrolase (type 1)

m-csa:523 - palmitoyl[protein] hydrolase (type 2)

3.1.3.2

m-csa:43 - purple acid phosphatase

m-csa:558 - non-specific acid phosphatases (class A)

m-csa:454 - high molecular weight acid phosphatase

3.1.3.5

m-csa:812 - 5'-nucleotidase (mitochondrial)

m-csa:611 - 5'-nucleotidase (bacterial)

3.1.3.16

m-csa:406 - protein phosphatase 2B

m-csa:456 - dual-specificity phosphatase

m-csa:472 - serine/threonine-protein phosphatase 5

3.1.3.48

m-csa:47 - protein-tyrosine-phosphatase non-receptor class

m-csa:469 - protein-tyrosine-phosphatase non-receptor type 1

m-csa:462 - low molecular weight phosphotyrosine protein phosphatase

3.1.11.3

m-csa:836 - exodeoxyribonuclease (lambda-induced)

m-csa:549 - exodeoxyribonuclease (lambda-induced)

3.1.21.1

m-csa:41 - deoxyribonuclease I

m-csa:791 - colicin-E9

3.1.21.4

m-csa:446 - type II site-specific deoxyribonuclease, Cfr10I/Bse634I

m-csa:495 - type II site-specific deoxyribonuclease, BglI

m-csa:897 - type II site-specific deoxyribonuclease PvuII

m-csa:927 - type II site-specific deoxyribonuclease FokI

3.1.27.3

m-csa:414 - ribonuclease T1

m-csa:459 - guanyl-specific ribonuclease Sa

3.1.27.5

m-csa:164 - ribonuclease 1

m-csa:693 - ribonuclease 4

3.2.1.4

m-csa:559 - cellulase (GH9)

m-csa:561 - cellulase (GH45)

m-csa:560 - cellulase (GH12)

3.2.1.8

m-csa:432 - endo-1,4-beta-xylanase (glycosyl hydrolase 11 family)

m-csa:548 - endo-1,4-beta-xylanase (glycosyl hydrolase 10)

3.2.1.14

m-csa:475 - chitinase (GH19, Class II)

m-csa:478 - chitinase (GH18, Class II)

m-csa:817 - chitinase (GH18, class II)

m-csa:819 - chitinase C

3.2.1.17

m-csa:203 - lysozyme (glycosyl hydrolase 22 family)

m-csa:774 - lysozyme (glycosyl hydrolase 25 family)

m-csa:921 - lysozyme (glycosyl hydrolase 24 family)

3.2.1.18

m-csa:835 - exo-alpha-sialidase (GH33 Family)

m-csa:828 - exo-alpha-sialidase (GH34 Family)

3.2.1.58

m-csa:831 - glucan 1,3-beta-glucosidase

m-csa:790 - glucan 1,3-beta-glucosidase

3.2.1.73

m-csa:400 - licheninase (glycosyl hydrolase 17 family)

m-csa:924 - licheninase (glycosyl hydrolase 16 family)

3.2.1.91

m-csa:444 - cellulose 1,4-beta-cellobiosidase (non-reducing end)

m-csa:440 - cellulose 1,4-beta-cellobiosidase (non-reducing end)

3.2.2.1

m-csa:39 - inosine-uridine preferring nucleoside hydrolase

m-csa:471 - inosine, adenosine, and guanine nucleoside hydrolase

3.4.11.19

m-csa:676 - D-stereospecific aminopeptidase (peptidase S58 family)

m-csa:782 - D-stereospecific aminopeptidase (peptidase S12 family)

3.4.19.12

m-csa:789 - ubiquitinyl hydrolase 1 (peptidase C19 type)

m-csa:597 - ubiquitinyl hydrolase 1 (peptidase C12 type)

m-csa:830 - ubiquitinyl hydrolase 1 (peptidase C30 type)

3.4.22.28

m-csa:477 - picornain 3C

m-csa:805 - staphopain

m-csa:763 - picornain 3C

3.5.1.2

m-csa:301 - glutaminase

m-csa:435 - carbamoyl-phosphate synthase (glutamine-hydrolysing)

3.5.1.11

m-csa:241 - penicillin amidase (peptidase C59 family)

m-csa:841 - penicillin amidase (peptidase S45 family)

3.5.1.77

m-csa:671 - N-carbamoyl-D-amino-acid hydrolase

m-csa:674 - N-carbamoyl-D-amino-acid hydrolase

3.5.2.6

m-csa:2 - beta-lactamase (Class A)

m-csa:15 - beta-lactamase (Class B1)

m-csa:16 - beta-lactamase (Class B1)

m-csa:210 - beta-lactamase (Class D)

m-csa:257 - beta-lactamase (Class C)

m-csa:258 - beta-lactamase (Class B1)

3.5.4.1

m-csa:710 - cytosine deaminase (bacterial)

m-csa:636 - cytosine deaminase (yeast)

3.6.3.14

m-csa:178 - H+-transporting two-sector ATPase (F-type, mitochondrial)

m-csa:507 - H+-transporting two-sector ATPase (F-type, bacterial)

m-csa:508 - H+-transporting two-sector ATPase (V-type)

3.6.4.12

m-csa:780 - DNA helicase

m-csa:833 - DNA helicase

3.6.5.-

m-csa:533 - G-protein alpha subunit, group I (GTPase)

m-csa:628 - small GTPase superfamily, ARF type

3.8.1.5

m-csa:415 - haloalkane dehalogenase (subfamily 1)

m-csa:467 - haloalkane dehalogenase (subfamily 2)

4.1.1.17

m-csa:860 - ornithine decarboxylase

m-csa:937 - ornithine decarboxylase

4.1.1.39

m-csa:797 - ribulose-bisphosphate carboxylase (type II)

m-csa:907 - ribulose-bisphosphate carboxylase (type I)

4.1.1.50

m-csa:225 - adenosylmethionine decarboxylase (prokaryotic)

m-csa:575 - adenosylmethionine decarboxylase (eukaryotic)

4.1.2.13

m-csa:52 - fructose-bisphosphate aldolase (Class II)

m-csa:222 - fructose-bisphosphate aldolase (Class I)

4.1.99.12

m-csa:683 - 3,4-dihydroxy-2-butanone-4-phosphate synthase

m-csa:648 - 3,4-dihydroxy-2-butanone-4-phosphate synthase

4.2.1.1

m-csa:216 - carbonate dehydratase (alpha class)

m-csa:517 - carbonate dehydratase (beta class)

m-csa:516 - carbonate dehydratase (gamma class)

4.2.1.8

m-csa:960 - mannonate dehydratase

m-csa:963 - mannonate dehydratase (uxuA)

4.2.1.10

m-csa:54 - 3-dehydroquinate dehydratase (type I)

m-csa:55 - 3-dehydroquinate dehydratase (type II)

4.2.1.59

m-csa:10 - 3-hydroxydecanoyl-[acyl-carrier-protein] dehydratase

m-csa:947 - 3-hydroxyacyl-[acyl-carrier-protein] dehydratase FabZ

4.2.1.158

m-csa:502 - galactarate dehydratase (D-threo-forming)

m-csa:503 - galactarate dehydratase (type III)

4.2.2.2

m-csa:184 - pectate lyase L (polysaccharide lyase 9 family)

m-csa:509 - pectate lyase

m-csa:896 - pectate lyase C (polysaccharide lyase 1 family)

4.2.99.18

m-csa:185 - 8-oxoguanine DNA-glycosylase (type-1 OGG1 family)

m-csa:510 - 8-oxoguanine DNA-glycosylase (ExoA family)

m-csa:888 - 8-oxoguanine DNA-glycosylase (Nth/MutY family)

4.3.1.17

m-csa:186 - L-serine ammonia-lyase

m-csa:956 - L-serine ammonia-lyase

4.4.1.5

m-csa:32 - lactoylglutathione lyase

m-csa:359 - lactoglutathione lyase

5.-.-.-

m-csa:372 - NikJ, nikkomycin biosynthesis protein P1

m-csa:503 - galactarate dehydratase (type III)

5.1.1.13

m-csa:335 - aspartate racemase (CC-type)

m-csa:388 - aspartate racemase (CT type)

5.2.1.8

m-csa:189 - peptidylprolyl isomerase (cyclophilin-type)

m-csa:362 - prolyl cis-trans isomerase (FKBP-type)

m-csa:511 - peptidylprolyl isomerase (PpiC-type)

5.3.1.8

m-csa:880 - mannose-6-phosphate isomerase (type I)

m-csa:736 - mannose-6-phosphate isomerase

5.3.3.8

m-csa:341 - 3,2-trans-enoyl-CoA isomerase (mitochondrial)

m-csa:499 - 3,2-trans-enoyl-CoA isomerase (peroxisomal)

5.3.4.1

m-csa:191 - protein disulfide-isomerase (eukaryotic)

m-csa:512 - protein disulfide-isomerase (DsbC)

5.4.99.5

m-csa:81 - chorismate mutase (AroQ)

m-csa:474 - chorismate mutase (AroH)

5.5.1.1

m-csa:269 - muconate cycloisomerase (syn)

m-csa:959 - muconate cycloisomerase (anti)

5.99.1.2

m-csa:64 - DNA topoisomerase (type III)

m-csa:232 - DNA topoisomerase (type IB)

m-csa:366 - DNA topoisomerase I (type 1A)

5.99.1.3

m-csa:745 - DNA topoisomerase (ATP-hydrolysing)

m-csa:744 - DNA topoisomerase (ATP-hydrolysing)

6.1.1.11

m-csa:884 - serine---tRNA ligase

m-csa:885 - Serine-tRNA ligase (archaeal)

6.3.2.3

m-csa:199 - glutathione synthase (prokaryotic)

m-csa:498 - glutathione synthase (eukaryotic)

2. List of M-CSA Entries with the same reference UniProt ID

P51659

m-csa:461 - enoyl-CoA hydratase

m-csa:470 - (3R)-hydroxyacyl-CoA dehydrogenase

P9WJ61

m-csa:954 - isotuberculosinol synthase

m-csa:955 - tuberculosinol synthase

O59791

m-csa:330 - serine racemase

m-csa:956 - L-serine ammonia-lyase

P22515

m-csa:307 - E1 ubiquitin-activating enzyme

m-csa:939 - E2 ubiquitin-conjugating enzyme

P11586

m-csa:385 - formyltetrahydrofolate synthetase

m-csa:389 - methylenetetrahydrofolate dehydrogenase (NADP+)

m-csa:458 - methenyltetrahydrofolate cyclohydrolase

P97852

m-csa:461 - enoyl-CoA hydratase

m-csa:470 - (3R)-hydroxyacyl-CoA dehydrogenase

O81959

m-csa:373 - Vanadium-dependent bromoperoxidase (organic sulfide oxidation)

m-csa:374 - Vanadium-dependent bromoperoxidase (organic molecule bromination)

P06104

m-csa:307 - E1 ubiquitin-activating enzyme

m-csa:939 - E2 ubiquitin-conjugating enzyme

P81701

m-csa:373 - Vanadium-dependent bromoperoxidase (organic sulfide oxidation)

m-csa:374 - Vanadium-dependent bromoperoxidase (organic molecule bromination)

m-csa:386 - Vanadium-dependent bromoperoxidase (HOBr Formation)

3. Data checks and automation in curator pages

The curator part of the website is organized as a set of pages where each one is roughly associated with the editing of one table of the database. Each page contains one or more forms that check the data for errors and automate some of the data entry. Some of these checks enforce rules at the database level like assuring that essential fields are not left blank – such as the enzyme name, or the entry description – and that fields that should be unique, like UniProtKB or ChEBI ids, remain so. Whenever possible, we also use selection fields instead of free text, to minimize input errors. For example, catalytic residues are selected from a list of residues taken from the PDB structure chosen as reference. Automation facilitates curation and is also useful to minimize the introduction of errors. We use automated forms when adding information relative to UniProtKB, PDB, ChEBI, and literature references. In these cases, the curator only needs to provide the main identifier for that element. The form checks if the identifier exists, and then retrieves the information required from the appropriate website. In the case of UniProtKB, for example, the form retrieves the enzyme name, a brief description, the sequence, and all associated PDBs. For literature references, both DOI and PubMed IDs can be used as identifiers.

4. Other examples from the statistics pages

Figure SI.1 shows the number of times a residue appears in the database as having a catalytic role in detailed mechanism entries. Charged residues are the most represented followed by cysteine and the polar tyrosine and serine. A further decomposition of the catalytic role of histidine is shown in figure SI.2. One can see that the most common role for histidine is as a *hydrogen bond donor and/or acceptor*, which are categorized as interaction roles. In terms of chemical active roles, histidine is a common *proton donor and acceptor*, and, among the spectator roles, can also act as an *electrostatic stabiliser*. Other plots on the statistics pages show the same information for other residues and organized by roles. There are also plots with the count of components and types of bond changes in the dataset.

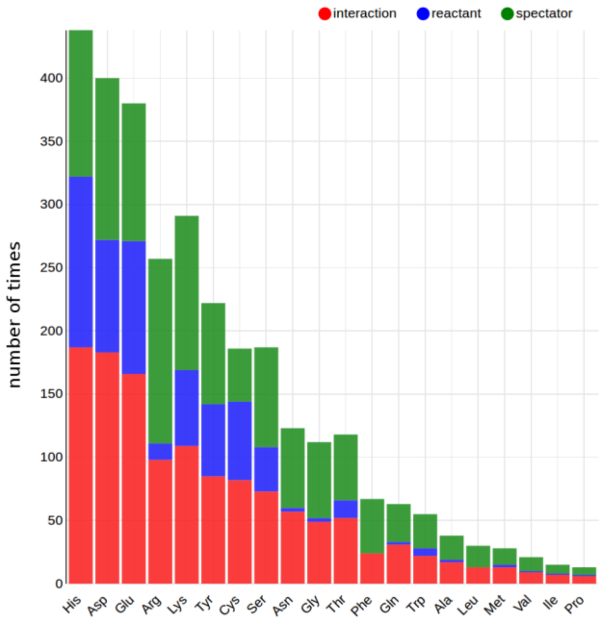

Figure SI.1 - Number of times each amino acid appears annotated with a catalytic role type in the current M-CSA dataset at the overall mechanism level.

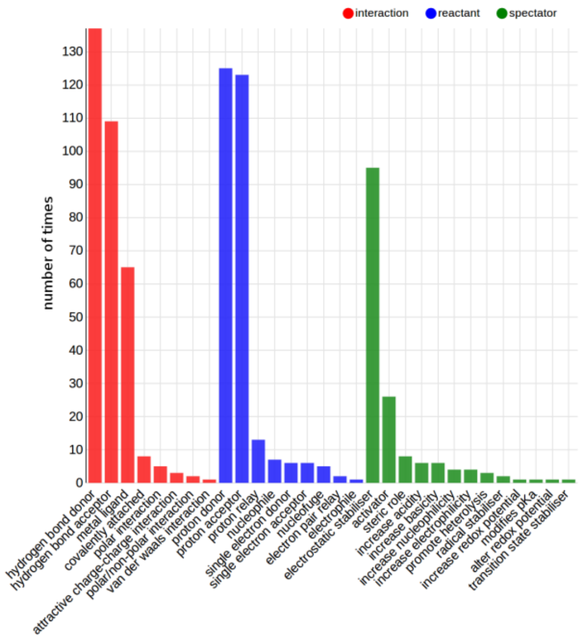

Figure SI.2 - Catalytic roles of His as annotated in the current M-CSA dataset. Roles are coloured by type: red - interaction, blue - reactant, green – spectator.

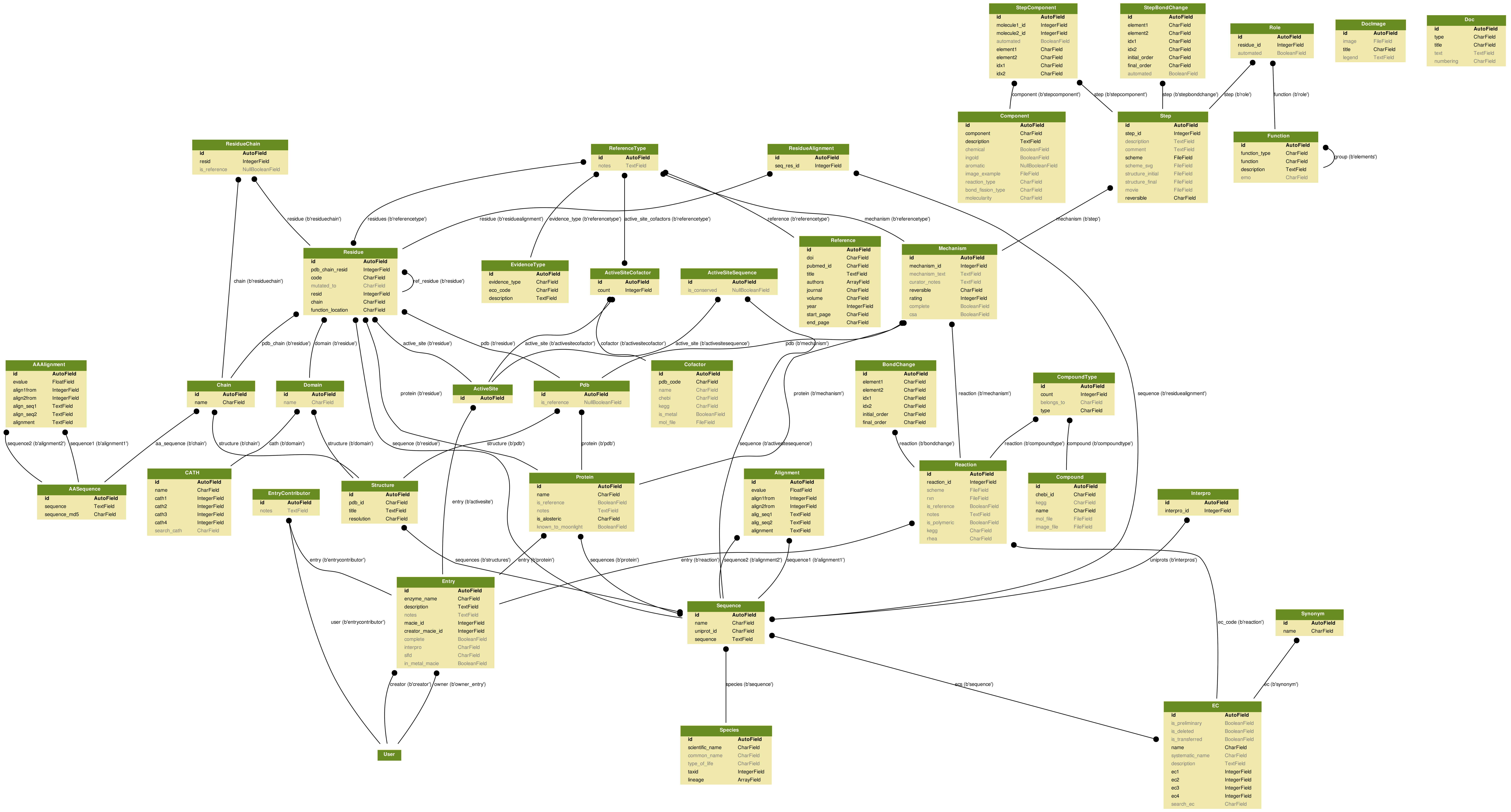

StepComponent
id AutoField
molecule1_id IntegerField
molecule2_id IntegerField
automated BooleanField
element1 CharField
element2 CharField
idx1 CharField
idx2 CharField
StepBondChange
id AutoField
element1 CharField
element2 CharField
idx1 CharField
idx2 CharField
initial_order CharField
final_order CharField
automated BooleanField
Role
id AutoField
residue_id IntegerField
automated BooleanField
DocImage
id AutoField
image FileField
title CharField
legend TextField
Doc
id AutoField
type CharField
title CharField
text TextField
numbering CharField
component (b'stepcomponent')
step (b'stepcomponent')
step (b'stepbondchange')
step (b'role')
function (b'role')
Component
id AutoField
component CharField
description TextField
chemical BooleanField
ingold BooleanField
aromatic NullBooleanField
image_example FileField
reaction_type CharField
bond_fission_type CharField
molecularity CharField
Step
id AutoField
step_id IntegerField
description TextField
comment TextField
scheme FileField
scheme_svg FileField
structure_initial FileField
structure_final FileField
movie FileField
reversible CharField
Function
id AutoField
function_type CharField
function CharField
description TextField
emo CharField
group (b'elements')
ResidueChain
id AutoField
resid IntegerField
is_reference NullBooleanField
ReferenceType
id AutoField
notes TextField
ResidueAlignment
id AutoField
seq_res_id IntegerField
residue (b'residuechain')
residues (b'referencetype')
residue (b'residuealignment')
evidence_type (b'referencetype')
active_site_cofactors (b'referencetype')
reference (b'referencetype')
mechanism (b'referencetype')
mechanism (b'step')
Residue
id AutoField
pdb_chain_resid IntegerField
code CharField
mutated_to CharField
resid IntegerField
chain CharField
function_location CharField
ref_residue (b'residue')
EvidenceType
id AutoField
evidence_type CharField
eco_code CharField
description TextField
ActiveSiteCofactor
id AutoField
count IntegerField
ActiveSiteSequence
id AutoField
is_conserved NullBooleanField
Reference
id AutoField
doi CharField
pubmed_id CharField
title TextField
authors ArrayField
journal CharField
volume CharField
year IntegerField
start_page CharField
end_page CharField
Mechanism
id AutoField
mechanism_id IntegerField
mechanism_text TextField
curator_notes TextField
reversible CharField
rating IntegerField
complete BooleanField
csa BooleanField
chain (b'residuechain')
pdb_chain (b'residue')
domain (b'residue')
active_site (b'residue')
pdb (b'residue')
active_site (b'activesitecofactor')
cofactor (b'activesitecofactor')
active_site (b'activesitesequence')
pdb (b'mechanism')
AAAlignment
id AutoField
evalue FloatField
align1from IntegerField
align2from IntegerField
align_seq1 TextField
align_seq2 TextField
alignment TextField
Chain
id AutoField
name CharField
Domain
id AutoField
name CharField
ActiveSite
id AutoField
Pdb
id AutoField
is_reference NullBooleanField
Cofactor
id AutoField
pdb_code CharField
name CharField
chebi CharField
kegg CharField
is_metal BooleanField
mol_file FileField
BondChange
id AutoField
element1 CharField
element2 CharField
idx1 CharField
idx2 CharField
initial_order CharField
final_order CharField
CompoundType
id AutoField
count IntegerField
belongs_to CharField
type CharField
protein (b'residue')
protein (b'mechanism')
reaction (b'mechanism')
sequence (b'residuealignment')
sequence2 (b'alignment2')
sequence1 (b'alignment1')
aa_sequence (b'chain')
structure (b'chain')
cath (b'domain')
structure (b'domain')
sequence (b'residue')
structure (b'pdb')
protein (b'pdb')
sequence (b'activesitesequence')
reaction (b'bondchange')
reaction (b'compoundtype')
compound (b'compoundtype')
AASequence
id AutoField
sequence TextField
sequence_md5 CharField
CATH
id AutoField
name CharField
cath1 IntegerField
cath2 IntegerField
cath3 IntegerField
cath4 IntegerField
search_cath CharField
EntryContributor
id AutoField
notes TextField
Structure
id AutoField
pdb_id CharField
title TextField
resolution CharField
Protein
id AutoField
name CharField
is_reference BooleanField
notes TextField
is_alosteric CharField
known_to_moonlight BooleanField
Alignment
id AutoField
evalue FloatField
align1from IntegerField
align2from IntegerField
alig_seq1 TextField
alig_seq2 TextField
alignment TextField
Reaction
id AutoField
reaction_id IntegerField
scheme FileField
rxn FileField
is_reference BooleanField
notes TextField
is_polymeric BooleanField
kegg CharField
rhea CharField
Compound
id AutoField
chebi_id CharField
kegg CharField
name CharField
mol_file FileField
image_file FileField
Interpro
id AutoField
interpro_id IntegerField
entry (b'activesite')
entry (b'entrycontributor')
sequences (b'structures')
entry (b'protein')
sequences (b'protein')
entry (b'reaction')
sequence2 (b'alignment2')
sequence1 (b'alignment1')
uniprots (b'interpros')
Entry
id AutoField
enzyme_name CharField
description TextField
notes TextField
macie_id IntegerField
creator_macie_id IntegerField
complete BooleanField
interpro CharField
sifd CharField
in_metal_macie BooleanField
Sequence
id AutoField
name CharField
uniprot_id CharField
sequence TextField
Synonym
id AutoField
name CharField
user (b'entrycontributor')
ec_code (b'reaction')
creator (b'creator')
owner (b'owner_entry')
species (b'sequence')
ecs (b'sequence')
ec (b'synonym')
User
Species
id AutoField
scientific_name CharField
common_name CharField
type_of_life CharField
taxid IntegerField
lineage ArrayField
EC
id AutoField
is_preliminary BooleanField
is_deleted BooleanField
is_transferred BooleanField
name CharField
systematic_name CharField
description TextField
ec1 IntegerField
ec2 IntegerField
ec3 IntegerField
ec4 IntegerField
search_ec CharField
